# The exploration of glucocorticoid pathway based on disease severity in COVID-19 patients

**DOI:** 10.1016/j.heliyon.2023.e23579

**Published:** 2023-12-10

**Authors:** Gestina Aliska, Andani Eka Putra, Fenty Anggrainy, Mutia Lailani

**Affiliations:** a Department of Pharmacology and Therapeutics, Faculty of Medicine, Universitas Andalas, Padang, 25176, Indonesia; b Centre for Diagnostic and Research on Infectious Disease (PDRPI), Faculty of Medicine, Universitas Andalas, Padang, Indonesia; c Department of Microbiology, Faculty of Medicine, Universitas Andalas, Padang, 2517, Indonesia; d Department of Pulmonology and Respiratory Medicine, Faculty of Medicine, Universitas Andalas, Padang, 2517, Indonesia; e Department of Physiology, Faculty of Medicine, Universitas Andalas, Padang, 2517, Indonesia; f Department of Clinical Pharmacology, Dr. M. Djamil General Hospital, Padang, Indonesia

**Keywords:** Cytokine, Coronavirus, Glucocorticoid-induced leucine zipper, Glucocorticoid receptor, Inflammation, Sequencing

## Abstract

Systemic inflammation is a hallmark of Coronavirus Disease 2019 (COVID-19) and is the key to the pathophysiology of its severe cases with host cytokine involvement. Glucocorticoids can moderate this inflammatory effect due to receptor binding (NRC31-the gene encoded), influencing the expression of effector genes and pro-inflammatory cytokines. Another important pathway in the processes of the immune and inflammatory responses is nuclear factor-κB (NF-κB) signaling (NFKBIA-the gene encoded). We aimed to explore the expression of genes in the glucocorticoid pathway in mild and severe COVID-19. We performed a cross-sectional, observational study on COVID-19 cases, assessing the expression of RNA in white blood cells. The Illumina® platform was used for RNA sequencing, and FASTQ data were quality-checked with Multiqc. The raw data were analyzed using CLC Genomics Workbench®. Our study included 23 patients with severe COVID-19 and 21 patients with mild COVID-19 with an average age of 49.9 ± 18.2 years old. The NR3C1 and NFKBIA expressions did not show a significantly significant difference between groups (log2 fold change 0.5, p = 0.1; 0.82, p = 0.09). However, the expressions of TSC22D3, DUSP-1, JAK-1 and MAPK-1 were significantly higher in mild cases (log2 fold change 1.3, p < 0.001; 2.6, p < 0.001; 0.9, p < 0.001; 1.48, p-value<0.001; respectively). Furthermore, the TNF, IL-1β, and IL-6 expressions were significantly lower in mild cases (log2 fold change 4.05, p < 0.001; 3.33, p < 0.001; 6.86, p < 0.001; respectively). In conclusion, our results showed that although the NRC31 and NFKBIA expressions did not show a statistically significant difference between groups, the expression of TSC22D3 was higher in mild cases. These results highlight the importance of effector genes, specifically TSC22D3, in combatting systemic inflammation. Our recent findings have the potential to lead to the identification of novel pharmacological targets that could prove to be vital in the fight against diseases associated with inflammation.

## Highlights

• Our study demonstrated that the expression of the TSC22D3 gene was enhanced in mild COVID-19 despite no significant differences in expressions of NR3C1 and NFKBIA compared to severe cases.
• The expressions of other genes that are not included in the potential main pathway of steroids' anti-inflammatory effects, yet affected by steroid binding and its receptors, i.e., DUSP-1, JAK-1 and MAPK-1, were dramatically higher in mild COVID-19 patients.
• Our current finding elucidates the functional importance of the effector genes, particularly TSC22D3, which may lead us to discover novel pharmacological targets for systemic inflammation.

## 1 Introduction

**Fig. 1:**
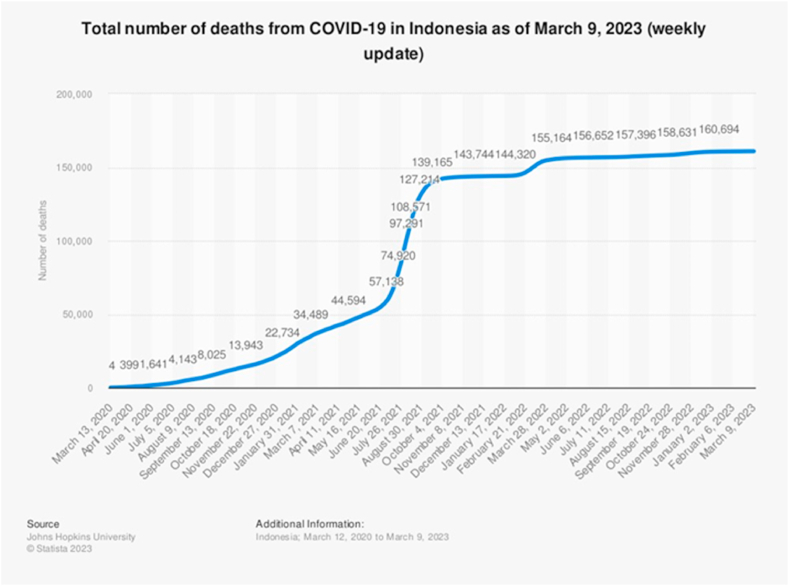
Total number of deaths from COVID-19 in Indonesia from March 2020 to March 2023. This figure showed more than 160,000 deaths in the end of the pandemic.

Source: Johns Hopkins University (2023, March) Total number of deaths from COVID-19 in Indonesia as of March 9, 2023 (weekly update) [Infographic]. Statista. https://www.statista.com/statistics/1103816/indonesia-covid-19-number-of-deaths/

The emergence of SARS-CoV-2 infection presents varying implications on the health outcomes of afflicted individuals [1,2]. Patients with COVID-19 may experience systemic inflammation, which plays a critical role in the pathophysiology of the infection. In particular, host cytokines are known to be involved in moderate and severe cases [3]. Several factors including the pro-inflammatory cytokine levels could lead to mortality [4]. In Indonesia, the number of deaths significantly increased from July until October 2021 (Fig. 1.) [5]. Low-dose corticosteroids reduced mortality in COVID-19 intensive care unit patients, but they have pleiotropic effects on patients and provide no benefit in mild cases [[6], [7], [8], [9]]. The binding of a glucocorticoid to its receptor (Glucocorticoid receptor, GR/Nuclear receptor subfamily 3 group C member 1, NR3C1) may repress inflammatory gene transcription through direct protein synthesis-independent processes (*trans*-repression) or by activating transcription of multiple anti-inflammatory/repressive factors (transactivation) [10].

Furthermore, previous studies have revealed that the immunosuppressive effects of steroids are primarily accomplished by upregulating the activity of NF-kappa-B inhibitor (NFKBIA) and triggering the activation of Glucocorticoid-induced leucine zipper (GILZ), which is encoded by the TSC22D3 gene. GILZ is a protein that inhibits T-cell receptor (TCR)-induced interleukin-2/interleukin-2 receptor expression and NF-κB activity. It responds to dexamethasone by increasing its expression but is suppressed when T-cell receptors are activated. GILZ regulates its own expression through a feedback loop linked to TCR activity [11,12].

Moreover, several other genes are affected by steroid binding and its receptors, including Dual specificity phosphatase 1 (DUSP-1), Janus kinase 1 (JAK-1), and Mitogen-activated protein kinase 1 (MAPK-1) [13,14]. By activating this pathway, it is expected that the production of proinflammatory cytokines such as Interleukin 6 (IL-6), Interleukin 1 Beta (IL-1β), and tumor necrosis factor (TNF) can be suppressed. The involvement of these cytokines in COVID-19 is well-described, hence the importance of modulating their expression [[15], [16], [17]].

Due to the limited number of research available regarding the glucocorticoid pathways in patients afflicted with COVID-19, coupled with the varying degrees of disease severity, we decided to conduct an exploration on this subject matter. This study investigated the expression of the glucocorticoid receptor (NR3C1); glucocorticoid pathways such as NFKBIA and TSC22D3; several markers for inflammation, such as DUSP-1, JAK-1, MAPK-1, TNF, IL-1β, and IL-6 in mild and severe cases of SARS-CoV-2 infection. To the best of our knowledge, this study is the first to examine the glucocorticoid pathway in relation to disease severity in COVID-19 patients.

## 2 Materials and methods

### 2.1 Study population

From October 2021 to February 2022, we conducted a cross-sectional, observational study on COVID-19 cases in several West Sumatra Province, Indonesia, referral hospitals. Our research was performed using Human Biological Material (HBM), mainly whole blood samples from adult patients older than 18 years old with corticosteroids as the only anti-inflammatory medications. Samples from pregnant patients were excluded.

After receiving ethical approval, all specimens were screened. In this study, we included 44 whole blood specimens from COVID-19 patients. The informed consent from all patients for using their specimens in our study was obtained when the whole blood was collected as regular examinations. Laboratory and clinical data were taken from medical records.

### 2.2 Sample collections

The regular examination included the complete blood count test, obtaining 5 mL of EDTA-treated blood via venepuncture, and other tests at the Central Laboratory. An aliquot of 1.5 mL was saved and prepared for leukocyte isolation. A whole blood specimen was used to isolate peripheral white blood cells using a QIAMP® RNA Blood kit. The RNA quantity was determined using a Qubit® 4 Fluorometer. The isolated RNA was kept at −80 °C. The Illumina® Stranded mRNA Prep Ligation Kit was used to prepare all samples for library preparation. Following preparation, the samples were maintained at −20 °C. Nextseq 550 Illumina® was used for RNA-seq.

### 2.3 Bioinformatics analysis

The reference sequence hg38 was used to analyze gene expression. The FASTQ data from Illumina® had quality checking with Multiqc. The raw data were analyzed using CLC Genomics Workbench® 21 version 21.0.3.

Ethics, Consent, and Permissions.

The protocol for this study was approved by the Institutional Review Board of M. Djamil Hospital (No. 454/KEPK/2021). Before being recruited as a subject, patients and/or their legal representatives were provided with informed consent. Samples were obtained from specimen banks from COVID-19 patients in the hospital.

### 2.4 Statistical analysis

Statistical analysis of the characteristic data was performed by SPSS® version 20.0. The normality of the data was assessed by the Saphiro-wilk test. Numerical data were analyzed with an independent T-test or Mann-Whitney test. All categorical data were analyzed by Chi-square.

Meanwhile, the statistical analysis of the differences in the gene expressions between mild vs severe cases was automatically generated by CLC Genomics Workbench® 21 version 21.0.3. P-value of. <0.05 means statistically significant.

## 3 Results

A total of 44 patients were included in our analysis, consisting of 21 mild cases and 23 severe cases (Table 1). The age was significantly different between the groups. Moderate-severe patients were older than mild patients. However, the sex ratio was similar between groups.

**Table 1:** Patient's characteristics. The data show significant differences in age and leukocyte concentration between the groups. The other variables, including sex and other laboratory blood test results, show no differences.

Characteristics	Mild	Moderate-Severe	P value
Age (years, mean ± SD)	39.4 ± 17.2	58.7 ± 14.1	<0.001
Gender			
**Male (n, %)**	7 (35 %)	9 (37,5 %)	
**Female (n, %)**	13 (65 %)	15 (62,5 %)	0.864
Laboratory			
**Haemoglobin (g/dL, mean ± SD)**	13.1 ± 1.9	12.4 ± 2.5	0.318
**Haematocrit (%, median, min-max)**	41.4 ± 6.6	37.9 ± 7.6	0.108
**Leukocyte (10** [3]**/μl, median, min-max)**	5585 (3400–15,000)	10,315 (4480–21,320)	<0.001
**Thrombocyte (10** [3]**/μl, median, min-max)**	189,000 (134,000–561,000)	204,000 (24,000–561,000)	0.953

The glucocorticoid receptor (NR3C1) expression in mild vs. severe cases was 1.41 fold change, but it was statistically insignificant (Table 2). However, the expression of TSC22D3 was significantly higher in mild cases than in moderate-severe cases (2.47 fold change, p < 0.001). Moreover, we found that the expression of NFKBIA was not significantly different between the groups.

**Table 2:** Glucocorticoid receptor and its main pathway in COVID-19 in mild vs. severe cases of COVID-19. The table demonstrates that a significant difference of change was only found in the gene expression of TSC22D3, while the expression of the receptor, NR3C1, and the other gene, NFKBIA, showed no differences.

Name	Max group mean	Log₂ fold change	Fold change	P-value
NR3C1	3.23	0.5	1.41	0.1
NFKBIA	139.09	0.82	1.77	0.09
TSC22D3	116.51	1.3	2.747	<0.001

NR3C1: Nuclear receptor subfamily 3 group C member 1; NFKBIA: NF-kappa-B inhibitor; TSC22D3; TSC22 Domain Family Member 3, encoding the protein glucocorticoid-induced leucine zipper (GILZ).

**Fig. 2:**
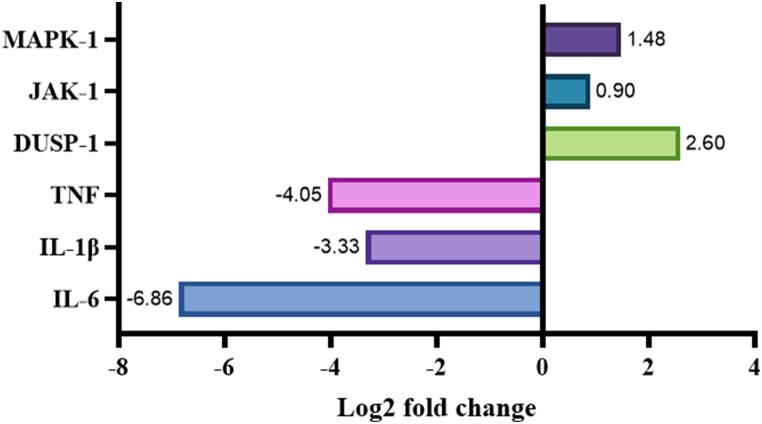
The RNA expression of inflammation marker in mild compared to severe cases of COVID-19. The anti-inflammatory markers i.e., MAPK-1, JAK-1, and DUSP-1, showed increased levels, meanwhile the pro-inflammatory markers i.e., TNF, IL-1β, and IL-6. showed decreased levels in the mild cases group. MAPK-1: mitogen-activated protein kinase 1; JAK-1: janus kinase 1; DUSP-1: dual specificity phosphatase 1; TNF: tumor necrosis factor; IL-1β: interleukin 1 beta; IL-6: interleukin 6 (IL-6).

The other effector genes in GC pathways like DUSP-1, JAK-1, and MAPK-1 were higher in mild cases than severe cases, while the pro-inflammatory cytokines IL-6, IL-1β, and TNF were dramatically lower in mild cases than severe cases (Fig. 2.).

## 4 Discussion

In this study, we assessed the presence of a difference in the expression of a gene encoding glucocorticoid receptor, nuclear receptor subfamily 3 group C member 1 (NR3C1), as well as its effector genes, NFKBIA, TSC33D3, dual specificity phosphatase 1 (DUSP1), JAK-1 and MAPK-1, and the expression of cytokines, such as tumor necrosis factor (TNF), IL-6 and IL-1β in mild and severe COVID-19 patients. Our results demonstrated significant differences in TSC22D3, TNF, DUSP1, and other marker's expression between groups, yet NFKBIA and NR3C1 expression did not differ.

These findings are in line with our previous study demonstrating that the response to corticosteroids in moderate to severe COVID-19 patients is determined by the pre-existing levels of glucocorticoid receptors. The study demonstrated increased expressions of the glucocorticoid receptor (NR3C1) and the effector genes (NFKBIA and TSC22D3) in patients responsive to corticosteroids [13]. However, some of our research results contrasted with the findings reported in another study by Camblor et al., which found that certain variants in the NF‐κB genes (NFKB1, NFKBIA, and NFKBIZ) may increase the risk of developing severe COVID‐19. The study also revealed that the NFKBIZ gene significantly impacts mortality [18]. Interestingly, our study found that there were no differences in NRC31 and NFKBIA between COVID-19 patients with severe and mild conditions.

It is well known that the NR3C1 gene encodes glucocorticoid receptor (GR), which binds to both exogenous and endogenous glucocorticoids involved in various physiological functions, particularly inflammatory processes [19]. Interestingly, we did not find differences between the group's NR3C1 (rs41423247) expression. Our finding contrasts with a previous study of scRNA-seq data of COVID-19 patient bronchoalveolar lavage fluid to compare immunological response according to the patient's disease severity. The study demonstrated that NR3C1 expression was higher in mild COVID-19 cases and lower in severe cases [20].

Nevertheless, our finding is in accordance with a recent study determining the effect of NR3C1 gene variants on the course of the disease in patients with COVID-19 using the PCR-RFLP method. The study suggested that the presence of NR3C1 was not related to severe cases of COVID-19 yet associated with longer glucocorticoid therapy [21].

The presence of GR in lymphocytes as well as their related genes, have been well established, yet factors affecting their regulations are still poorly studied. Several factors have been identified as factors affecting the gene encoding GR, the NR3C1, including biopsychosocial factors such as lower economic status and negative emotional relationships that are interwoven among family members (family negative emotional climate) [22].

Other factors have also been identified in recent studies, such as cocaine addiction and posttraumatic stress disorder [23,24]. COVID-19 patients with well-response to steroid therapy have higher NR3C1 expression than the poor-response group [13]. Nonetheless, our results suggested that the severity of COVID-19 is unrelated to the gene's expression.

Furthermore, our findings showed that other genes, including TSC22D3, DUSP-1, JAK-1, and MAPK-1 expressions, were significantly elevated in mild COVID-19. The SARS-CoV-2 spike protein activates the phosphorylation pathway, which triggers TLR4 signaling. This leads to the activation of p38 MAPK and NF-κB. In addition, the spike protein also activates IRAK4 signaling, which induces cytokine storm, p38 MAPK, and NF-κB. It inhibits DUSPs and Wip1, which results in sustained p53 expression. JNK-microRNA-16 activation leads to Wip1 deficiency, causing a decrease in the deactivation of p53, followed by the induction of cytokines [25]. Moreover, the JAK/STAT pathway plays a crucial role in transmitting signals for cytokines and chemokines. However, studies have shown that it can also worsen the effects of COVID-19. When cytokine receptors activate JAK phosphorylation, it triggers the phosphorylation of STATs. These STATs then move into the nucleus to produce inflammatory mediators [26].

Recent developments in the understanding of COVID-19 show that the severity of the disease may be linked to cytokine release. Our study demonstrated that the expression of TNF in the leucocytes was higher in the severe COVID-19 cases compared to the mild cases. The result supported previous studies, thus suggesting that TNF is a potential biomarker to predict COVID-19 severity [27]. The high levels of TNF in severe COVID-19 are likely because of the activation of induced cell death and/or the redistribution of cells in tissue fields [28]. Moreover, a previous review study of thousands of COVID-19 patients in China about leukocyte changes and cytokine storm in mild versus severe cases confirmed that mild and severe conditions affected circulating leukocyte subsets and cytokine secretion, including TNF [29]. In accordance with our results, previous studies demonstrated that COVID-19 is associated with elevated levels of TNF, IL-1β, and IL-6 [30,31].

When macrophages encounter inflammatory triggers, they generate cytokines such as TNF, IL-1, and IL-6. Other cells like activated lymphocytes, endothelial cells, and fibroblasts can also produce a small number of cytokines. Furthermore, macrophages release various substances such as chemokines, leukotrienes, prostaglandins, and complements into the body. These substances can cause systemic effects and an acute inflammatory response, leading to septic shock and multi-organ failure [32]. Thus, managing cytokine release has been suggested as a way to potentially save severe COVID-19 patients.

Why the human immune response to COVID-19 varies so widely is still unclear. It is suggested that tracking the patient responses over time may provide a better understanding of this condition and has implications for predicting disease severity since the TNF expression is potentially related to the phase of infections [4,33].

In this study, our moderate-severe COVID-19 patients were older than the mild patients. Our results are in line with current findings that COVID-19 morbidity risk factors in adults include old age, male sex, pre-existing comorbidities, and ethnic disparities [4]. Aging affects the immune system, causing cytokine dysregulation and suppressing T-cell responses. This can lead to health complications, emphasizing the need to understand immune function in older patients. This phenomenon can lead to an exhausted composition of CD4^+^ and CD8^+^ T lymphocytes, further exacerbated by a specific cytokine environment. Regrettably, this suboptimal immune response may contribute to the challenges aged patients face in combating SARS-CoV-2 viruses [34]. Recent research has unveiled the presence of specific molecular pathways that are intrinsically linked to the ageing process and low-grade inflammation. These pathways encompass alterations in redox balance, an upsurge in senescent cells, the senescence-associated secretory phenotype (SASP), and a decline in the efficacy of autophagy, which can trigger inflammation [35].

However, we could not confidently confirm the relationship between the expression of the NR3C1 and its effector genes due to the limitations of our study. These limitations included the small size of the sample and the limited center participating in this research. Additionally, we noticed that our study population consisted of people with the same ethnicity from South-East Asia and that there was a statistically significant age difference between our cases and controls. Thus, we believe that our interesting findings should be further studied in the future in order to develop our understanding of the roles of NR3C1 and its related genes, particularly in COVID-19 conditions.

## 5 Conclusions

In summary, our analysis indicates that there was no significant difference in the expression levels of NRC31 and NFKBIA between the various groups studied. However, it is noteworthy that TSC22D3 levels were observed to be considerably higher in cases that presented with milder symptoms. These findings suggest a correlation between TSC22D3 expression and disease severity, potentially associated with inflammation. This work demonstrates the functional importance of effector genes, particularly TSC22D3, which may lead to the discovery of novel pharmacological targets for systemic inflammation. In contrast, our study did not reproduce previously reported associations between NR3C1 expression and its effector genes. This may be due to small sample size, limited participation, and age and ethnic differences among the study population. Therefore, we suggested that the fascinating findings we have uncovered warrant deeper exploration to expand our comprehension of the roles of NR3C1 and its related genes, especially with regard to COVID-19.

## Funding statement

This study was funded by the Research Fund from the Faculty of Medicine, 10.13039/501100014563Universitas Andalas.

## Data availability statement

Data will be made available on request.

## Ethical statement

The protocol for this study was approved by the Institutional Review Board of M. Djamil Hospital (No. 454/KEPK/2021). Before being recruited as a subject, patients and/or their legal representatives were provided with informed consent. Samples were obtained from specimen banks from COVID-19 patients in the hospital.

## CRediT authorship contribution statement

**Gestina Aliska:** Writing – original draft, Visualization, Methodology, Investigation, Funding acquisition, Formal analysis, Conceptualization. **Andani Eka Putra:** Writing – original draft, Validation, Supervision, Software, Resources, Data curation, Conceptualization. **Fenty Anggrainy:** Validation, Methodology, Investigation, Data curation, Conceptualization. **Mutia Lailani:** Writing – review & editing, Writing – original draft, Visualization, Project administration, Data curation.

## Declaration of competing interest

The authors declare that they have no known competing financial interests or personal relationships that could have appeared to influence the work reported in this paper.

Gestina Aliska reports financial support was provided by the Faculty of Medicine, Universitas Andalas. If there are other authors, they declare that they have no known competing financial interests or personal relationships that could have appeared to influence the work reported in this paper.
